# LONG-TERM IMPACT OF A LIFESTYLE INTERVENTION: THE ACTION FOR HEALTH IN DIABETES (LOOK AHEAD) TRIAL AND FOLLOW-UP

**DOI:** 10.1093/geroni/igad104.1697

**Published:** 2023-12-21

**Authors:** Denise Houston, Mark Espeland, Sara Espinoza

## Abstract

Mid-life weight loss and maintenance is often recommended as a means to improve health later in life, however its long-term benefits have not been rigorously established. The Look AHEAD randomized controlled trial compared an intensive lifestyle intervention (ILI) designed to induce and maintain a 7% weight loss through caloric restriction and increased physical activity with a control condition of diabetes support and education (DSE) in 5,145 individuals aged 45-76 years with type 2 diabetes and overweight/obesity over up to 11 years of follow-up beginning in 2001. Compared to DSE, ILI had greater initial improvements in cardiovascular disease risk factors; however, there were no overall differences in the incidence of the trial’s primary and secondary cardiovascular outcomes between the ILI and DSE groups. ILI did, though, have a positive impact on diabetes control and complications, depression, physical health–related quality of life, and health care use and costs. Subsequently, the Look AHEAD trial transitioned to an observational study in 2012 to assess the long-term benefits and potential harms of ILI, which continued for 10 years. This symposium will describe the design of the Look AHEAD trial and transition to the Look AHEAD Aging study, the most recent phone-only observational follow-up begun in 2022, and the long-term impact of the lifestyle intervention on sleep (Spira and colleagues), cognitive resilience (Espeland and colleagues), multi-morbidity and frailty assessed by the deficit accumulation model (Simpson and colleagues), and medical care utilization and costs (Davis and colleagues).

